# Fractures of the distal radius (Colles’ fracture)

**DOI:** 10.1590/S1516-31802007000300002

**Published:** 2007-05-03

**Authors:** João Carlos Belloti, João Baptista Gomes dos Santos, Álvaro Nagib Atallah, Walter Manna Albertoni

**Keywords:** Radius fractures, Epidemiology, Colles’ fracture, Questionnaires, Prospective studies, Fraturas do rádio, Epidemiologia, Fratura de Colles, Questionários, Estudos prospectivos

## Abstract

**CONTEXT AND OBJECTIVE:**

Although Colles’ fracture is a common clinical situation for the orthopedist, we did not find any information in the literature that would allow safe decision-making on the best treatment for each fracture type. The aim of the present study was to investigate Brazilian orthopedists’ opinions regarding the main aspects of treatments for Colles’ fracture.

**DESIGN AND SETTING:**

Cross-sectional study conducted during the 34^th^ Brazilian Congress of Orthopedics and Traumatology.

**METHODS:**

Five hundred questionnaires containing 12 items were randomly distributed to orthopedists who were attending the congress; 439 were filled out correctly and were considered in this study.

**RESULTS:**

The main factors in making decisions on interventions in fracture cases were whether the fracture was intra-articular, the existence of shortening of the distal radius and the patient's age. The classification method most used was Frykmann. The closed reduction method most used was manual reduction. The principal surgical interventions were percutaneous pinning (39%), external fixation (27%) and volar plate (19%). Most of the interviewees only used bone grafts for osseous gaps in special cases. The most frequent complications were impairment of joint mobility and residual pain.

**CONCLUSIONS:**

Brazilian orthopedists have concordant opinions regarding conservative treatment methods and the use of bone grafts. There were conflicting opinions regarding surgical treatment methods, classification types and complications.

## RESUMO

**CONTEXTO E OBJETIVO:**

Embora as fraturas de Colles sejam uma situação clínica comum para os ortopedistas, não encontramos na literatura elementos que permitam decidir com segurança sobre a melhor forma de tratamento para cada tipo dessas fraturas. O objetivo deste estudo foi verificar a conduta dos ortopedistas brasileiros quanto aos principais aspectos do tratamento das fraturas de Colles.

**TIPO DE ESTUDO E LOCAL:**

Estudo transversal, realizado durante o 34^o^ Congresso Brasileiro de Ortopedia e Traumatologia, São Paulo (SP).

**MÉTODOS:**

500 questionários, com 12 itens foram distribuídos aleatoriamente aos congressistas, sendo que 439 foram corretamente preenchidos e considerados no estudo.

**RESULTADOS:**

Os principais fatores para a decisão e opção da forma de tratamento foram o grau de acometimento articular, o encurtamento do rádio e a idade. O método de classificação das fraturas do rádio distal mais utilizada é o de Frikmann. Como métodos cirúrgicos, 39% dos entrevistados utilizam uma das três técnicas de pinagem percutânea, 27% utilizam o fixador externo e 19% utilizam osteossíntese com placa volar. Quanto à utilização de enxerto ósseo, a maioria dos entrevistados somente o utiliza em casos especiais. As complicações mais freqüentes foram a restrição do arco de movimento e a dor residual.

**CONCLUSÃO:**

A conduta do ortopedista brasileiro é concordante quanto à forma de tratamento conservador e à utilização de enxerto ósseo. Há conflito de opiniões quanto ao método de classificação das fraturas; aos métodos de tratamento cirúrgico e às complicações.

## INTRODUCTION

Although fractures of the distal extremity of the radius were first described by Colles in 1814, today there is still no robust scientific evidence to allow a definitive treatment algorithm to be devised. This is because of the complexity of these fractures with regard to patient type, associated lesions, trauma mechanisms and the several classification methods used.

We found in the literature a great number of papers on the several types and methods for treating these fractures, but without any definitions regarding the best treatment option for each fracture type. Among the publications presenting better levels of evidence, four systematic reviews of randomized clinical trials can be highlighted. Their topics were: “Surgical interventions for treating distal radial fractures in adults”,^1^ “Conservative interventions for treating distal radial fractures in adults”,^2^ “Anesthesia for treating distal radial fractures in adults”^3^ and “Rehabilitation for distal radial fractures in adults”.^4^

These studies concluded that there was no evidence that would allow decision-making regarding the best treatment, anesthesia and rehabilitation methods for each type of fracture of the distal radius. They recommended that new studies of good methodological quality should be conducted in order to supply better evidence for making decisions on the most appropriate treatment. There is still a great need to shorten the time taken for reintegrating patients into their habitual activities. There are also growing ethical and legal demands for cosmetic and functional results for patients.

## OBJECTIVE

To investigate how the orthopedists attending the 34^th^ Brazilian Congress of Orthopedics and Traumatology were treating Colles’ fracture.

## METHODS

During the 34^th^ Brazilian Congress of Orthopedics and Traumatology, which was held in the city of São Paulo (SP) in 2002, orthopedists who were attending the congress were invited to participate in the study. The participants filled out a questionnaire that had been drawn up previously, in which there were 12 objective multiple-choice questions that dealt with matters of relevance to treating Colles’ fracture.

To estimate the sample size, we took p = 50% (i.e. sample size as large as possible), since we did not know the proportion of orthopedists who were giving correct treatment. The analysis was performed by taking a sampling error of 7%, power of 80% and confidence interval of 95%, which resulted in a sample size of 400. The population was assumed to be infinite, because not every orthopedist attending the congress could be included in the target sample. Therefore, the sample size calculation was not related to the total number of participants.

Thus, 500 questionnaires were distributed randomly, during the lectures and talks of the congress. After immediate completion by the physician, the questionnaire was identified with a sequential number and filed. A total of 439 questionnaires were filled out correctly and were included in this study. The text of the questionnaire was as follows (our free translation from Portuguese):

“Questionnaire: 34^th^ Brazilian Congress of Orthopedics and Traumatology.

Discipline of Hand and Arm Surgery, Universidade Federal de São Paulo (Unifesp).

‘Protocol for evaluating clinical approaches to fractures of the distal radius’.

Dear congress attendee,

The purpose of this questionnaire is to investigate Brazilian orthopedists’ diagnostic and therapeutic methods, complications and results relating to clinical approaches to fractures of the distal radius. We kindly request your assistance by completing the items below. Only take into consideration fractures of the distal radius in patients over 40 years old, except for cases of fractures caused by avulsion and Barton's fracture. Thank you.

1. Of the items below, which do you consider more important in making treatment decisions (no more than three options)?
  - ( ) the patient's age
  - ( ) dorsal angulation of the fracture
  - ( ) shortening of the distal radius
  - ( ) intra-articular fracture
  - ( ) trauma mechanism
  - ( ) associated lesions
  - ( ) degree of osteoporosis
2. Which classification method do you use?
  - ( ) Melone
  - ( ) AO
  - ( ) Frykmann
  - ( ) Universal Classification
  - ( ) Fernandez
  - ( ) other
3. Which conservative treatment method do you use preferentially?
  - ( ) below-elbow plaster immobilization
  - ( ) above-elbow plaster immobilization
  - ( ) other type
4. When closed reduction of the fracture is necessary, which method do you use?
  - ( ) Manual reduction/outpatient/local anesthesia
  - ( ) Manual reduction/hospital/general or block anesthesia
  - ( ) Finger-trap traction/outpatient/local anesthesia
  - ( ) Finger-trap traction/hospital/general or block anesthesia
5. Which positions do you use for immobilization of the wrist (no more than three options)?
  - ( ) palmar flexion
  - ( ) dorsiflexion
  - ( ) ulnar deviation
  - ( ) radial deviation
  - ( ) pronation
  - ( ) supination
  - ( ) neutral (pronation/supination)
6. Which is your preferred surgical intervention (no more than two options)?
  - ( ) volar plate
  - ( ) dorsal plate
  - ( ) ulnar pinning
  - ( ) external fixation
  - ( ) intramedullary pinning
  - ( ) intrafocal pinning
  - ( ) associated methods: ___________
7. Do you use bone grafts or other substitution material?
  - ( ) Yes, in about 5% of surgical treatments
  - ( ) I never use them
  - ( ) Yes, in about 20% to 40% of surgical treatment
  - ( ) Yes, in more than 50% of surgical treatments
  - ( ) Yes, in all surgical treatments
8. Which is the most frequent complication in your conservative treatment (no more than two options)?
  - ( ) residual pain
  - ( ) impairment of joint mobility
  - ( ) reflex sympathetic dystrophy
  - ( ) cosmetic appearance
  - ( ) impairment of grip strength
  - ( ) malunion
9. Which is the most frequent complication in your surgical treatment (no more than two options)?
  - ( ) residual pain
  - ( ) impairment of joint mobility
  - ( ) reflex sympathetic dystrophy
  - ( ) malunion
  - ( ) impairment of grip strength
  - ( ) infection
  - ( ) tenosynovitis
10. Which associated injuries are most frequently diagnosed?
  - ( ) tendon injuries
  - ( ) median nerve injury
  - ( ) carpal ligament injuries
  - ( ) instability of the distal radioulnar joint
11. What is the usual statistical timeframe for fractures to heal and patients to return to their habitual activities, when conservative treatment is used?
  - ( ) 6 to 10 weeks
  - ( ) 10 to 12 weeks
  - ( ) 12 to 16 weeks
  - ( ) 16 to 20 weeks
  - ( ) more than 20 weeks
12. And when surgical treatment is used?
  - ( ) 6 to 10 weeks
  - ( ) 10 to 12 weeks
  - ( ) 12 to 16 weeks
  - ( ) 16 to 20 weeks
  - ( ) more than 20 weeks”

## RESULTS

**Figure 1:**
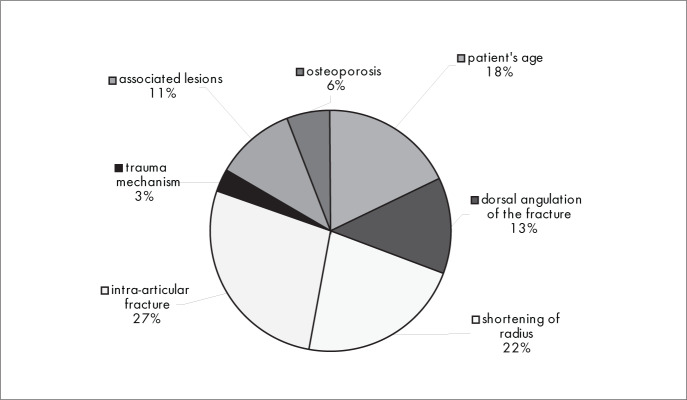
Results from question 1, about matters to consider in treatment choice.

**Figure 2:**
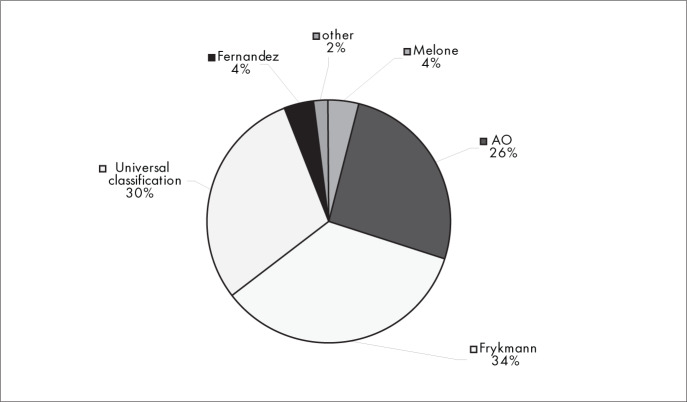
Results from question 2, about the classification method used.

The main factors in making decisions and choices regarding interventions (conservative or surgical treatment) of fractures were whether the fracture was intra-articular (27%), the existence of shortening of the distal radius (22%) and the patient's age (18%) (Figure 1). The classification method for fractures of the distal radius that was most used was Frykmann (34%), followed by the Universal Classification (30%) and the AO/ASIF classification (26%) (Figure 2).

**Figure 3:**
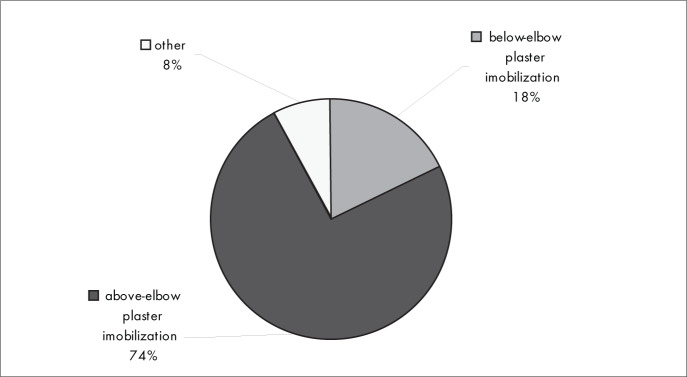
Results from question 3, about the preferred conservative method for Colles’ fracture.

Figure 3 shows that most of the orthopedists interviewed (74%) used above-elbow plaster immobilization in conservative treatment.

**Figure 4:**
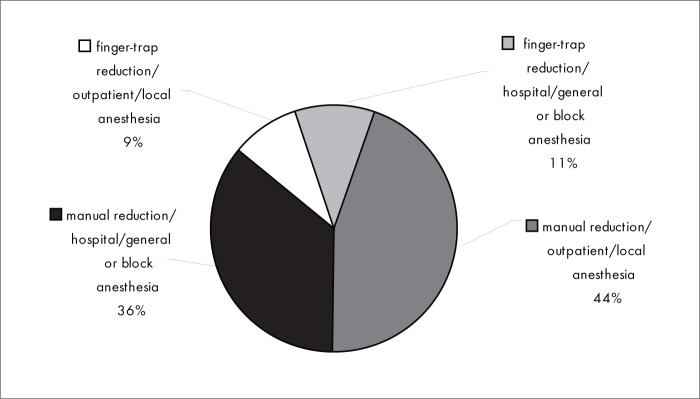
Results from question 4, about fracture reduction method.

Figure 4 shows that closed reduction method most used was manual reduction (80%). The anesthesia technique most used for closed reduction of the fracture was local anesthesia (53%). General or block anesthesia was used by 47% of the interviewees.

**Figure 5:**
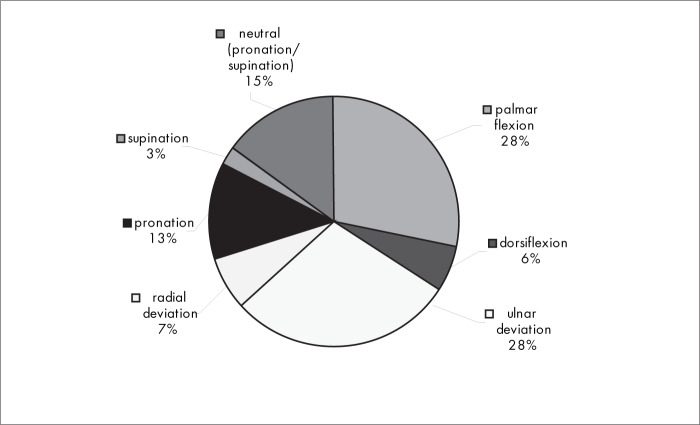
Results from question 5, about positions for immobilization.

Figure 5 shows that, with regard to the immobilization position for the wrist the palmar flexion, ulnar deviation and neutral (pronation/supination) positions were used most.

**Figure 6:**
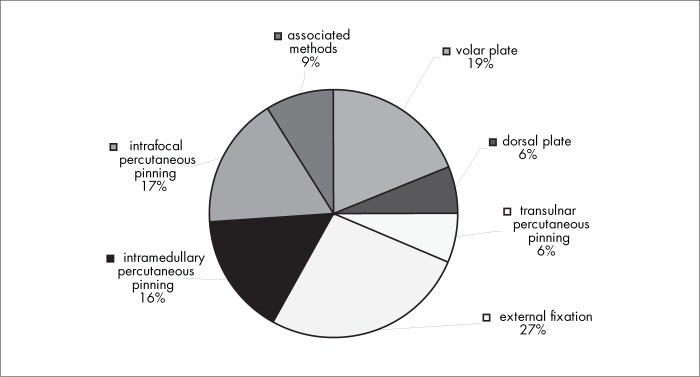
Results from question 6, about the choice of surgical technique.

Figure 6 shows that, among the surgical interventions, 39% of the interviewees used one of the three percutaneous pinning methods, 27% used external fixation and 19% used volar plates.

**Figure 7:**
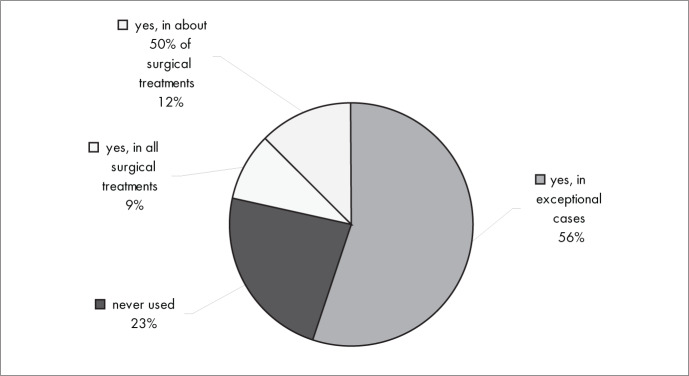
Results from question 7, about substitution materials for the bone.

Figure 7 shows that, with regard to the use of bone grafts or other substitution material for osseous gaps, most of the interviewees (56%) used them only in special cases, 23% never used them and 9% used them in all surgical treatments.

**Figure 8:**
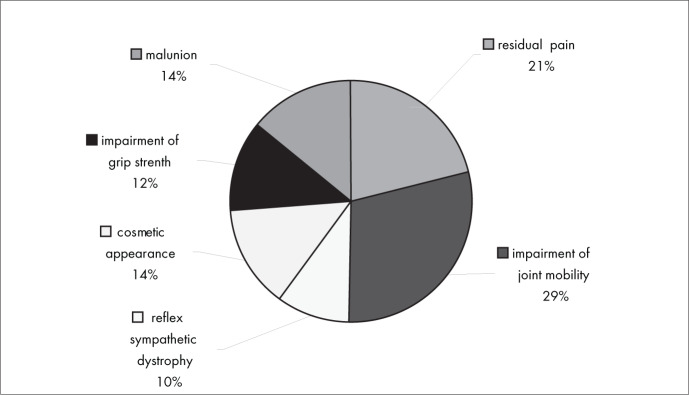
Results from question 8, about complications of conservative treatment.

Figure 8 shows that the most frequent complications in conservative treatment were impairment of joint mobility (29%) and residual pain (21%). The most unusual complications were cosmetic appearance (14%), malunion (14%), impairment of grip strength (12%) and reflex sympathetic dystrophy (10%).

**Figure 9:**
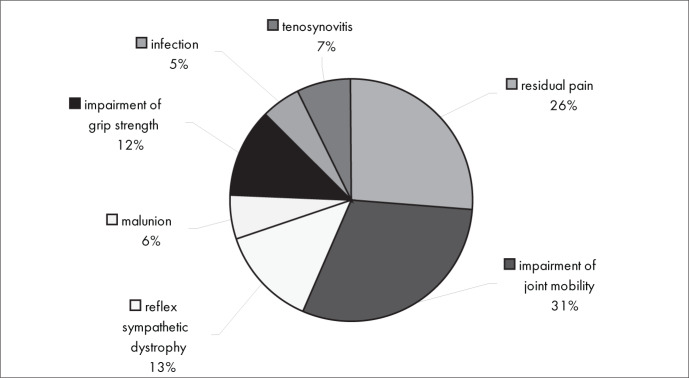
Results from question 9, about complications of surgical treatment.

Figure 9 shows that impairment of joint mobility (31%) and residual pain (26%) were also the most frequent complications in relation to surgical treatment. Infection (5%), malunion (6%) and tenosynovitis (7%) were the least frequent complications.

**Figure 10:**
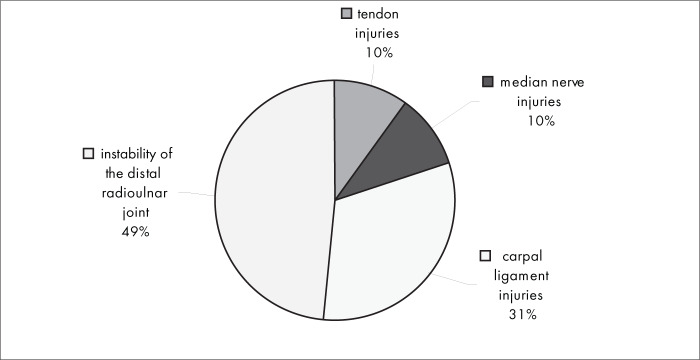
Results from question 10, about associated injuries.

Figure 10 shows that instability of the distal radioulnar joint (49%) was the associated injury that was most frequently diagnosed, followed by carpal ligament injuries (31%). Tendon injuries and median nerve injury were the least frequent ones (10%).

**Figure 11:**
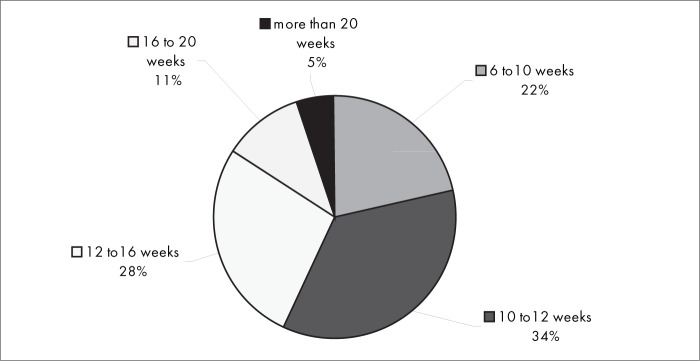
Results from question 11, about the time until the patient resumes activities with conservative treatment.

**Figure 12:**
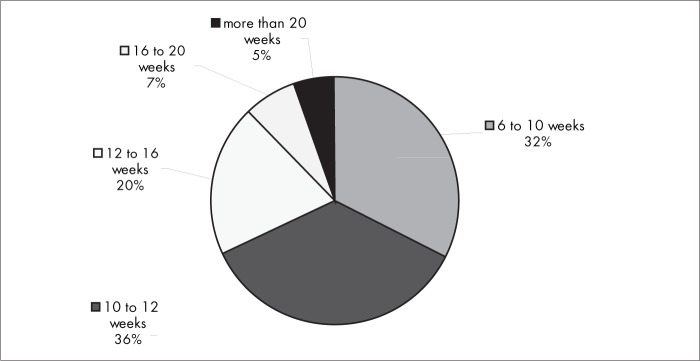
Results from question 11, about the time until the patient resumes activities with surgical treatment.

Figure 11 shows that, when conservative treatment was used, the usual statistical timeframe for fractures to heal and patients to return to their habitual activities was from 6 to 16 weeks for most of the interviewees (84%). The same was true for most of the interviewees (88%) regarding surgical treatment (Figure 12).

## DISCUSSION

Colles’ fracture is one of the most common among older white people. A prospective multicenter trial in the United Kingdom reported that the overall annual incidence of this fracture in patients aged 35 years and above was 9/10,000 among men and 37/10,000 among women.^5,6^

Most fractures of the distal radius result from low-energy trauma, such as falls from no more than the individual's own standing height, and their greater incidence among women reflects the loss of bone mass due to osteoporosis and the larger number of falls suffered by older women.

Although Colles’ fracture is a common clinical situation for the orthopedist, we did not find in the literature elements that would allow safe decision-making regarding the best treatment for each fracture type. However, over the last decade, better-quality scientific studies^1–4^ have been published, thus providing some evidence for treating these fractures.

In drawing up this questionnaire, we only dealt with Colles’ fractures, i.e. impacted fractures with dorsal angulation and radial shortening, plus fractures caused by avulsion or shearing. The objective was to evaluate the main features of treating these fractures, and also the most frequent complications and the prognosis.

The responses to the question on the three more important factors in making decisions and choices for treating these fractures (Figure 1) showed that most of the interviewees (27%) considered the main factors to be whether the fracture was intra-articular, the existence of shortening of the distal radius (22%) and the patient's age (18%). It is stated in the literature that the degree of restoration of the articular alignment is the main prognostic factor for the fracture;^7,8^ that the radial shortening that is seen on X-rays is considered to be one of the main elements denoting instability of the fracture;^9,10^ and that the patient's age reflects his or her potential for bone loss (instability).^7,10^ However, these are not excluding factors and they should be considered together in making decisions about the treatment.

The Frykmann classification method was used by the greatest percentage of the interviewees (34%), followed by the universal classification (30%) and the AO/ASIF classification (26%), whereas only 4% used the Fernandez classification (Figure 2). We did not find any definition in the literature regarding which classification method is the best, but several studies^7,11,12^ state that the classification methods that supply better parameters for treating these fractures are the universal, AO/ASIF and Fernandez classifications. Although the Frykmann classification is very widely used, it does not supply the minimum backing necessary for planning the treatment,^11^ since it essentially only supplies morphological data on the fracture and thus is not a recommended method.

The conservative treatment used by most of the interviewees was above-elbow plaster immobilization (74%) (Figure 3), and the principal immobilization positions were palmar flexion (28%), ulnar deviation (28%) and neutral (pronation/supination) (Figure 5).

In the systematic review “Conservative interventions for treating distal radial fractures in adults”,^2^ in which 33 randomized trials analyzed 3664 patients, there were descriptions of several studies comparing different conservative treatment methods, consisting of both external splintage (plaster of Paris casts, braces and bandages) and immobilization (above or below elbow, in supination, pronation or neutral positions, with palmar flexion or dorsiflexion). It was concluded that there was not enough evidence to decide which conservative treatment method was more appropriate for each type of fracture of the distal radius.

The closed reduction method that was used most was the manual reduction method (80%), and local anesthesia (for outpatients) was used by a majority of the interviewees (53%). However, 47% of the interviewees used general or block anesthesia, in hospital (Figure 4). These results are not concordant with the literature, where we found that about 80% of the patients with Colles’ fracture were treated as outpatients.^5,13^

In the systematic review: “Anesthesia for treating distal radial fractures in adults”,^3^ 18 randomized trials including 1200 patients were compared with regard to anesthesia methods: local anesthesia (hematoma block), intravenous regional anesthesia, brachial plexus nerve block and general anesthesia. There was no conclusive evidence on the best anesthesia method in relation to effectiveness, safety and influence on fracture reduction. However, some of the evidence indicated that local anesthesia (hematoma block) produced worse analgesia than did intravenous regional anesthesia, and thus it hinders fracture reduction.

There is no evidence favoring one method for closed reduction over another. A randomized trial published in 2002 compared the reduction methods of manual manipulation and finger-trap traction, among 223 patients with 225 fractures. It was shown that there was no statistically significant difference between these two reduction methods.^14^ The two surgical intervention methods preferred by the interviewees in our study were the techniques of percutaneous pinning (39%) and external fixation (27%) (Figure 6).

Substitute material was only used in exceptional cases by 56% of the interviewees, while 26% never used it. In the systematic review “Surgical interventions for treating distal radial fractures in adults”,^1^ with 44 randomized trials and 3193 patients, it was concluded that there was not enough evidence for most of the decisions needed for surgically treating fractures of the distal radius. Nonetheless, there was some favorable evidence supporting the use of external fixation and percutaneous pinning.

There were no significant differences in complications between conservative and surgical interventions. Residual pain and impaired joint mobility were the most frequent complications in both treatment types (Figures 8 and 9). Instability of the distal radioulnar joint was the associated injury that was most frequently diagnosed (Figure 10).

Among the complications from conservative intervention were residual pain, impaired joint mobility, malunion of the fracture and reflex sympathetic dystrophy. The most frequent complications from surgical treatment were related to plate synovitis, adhesions, infection and reflex sympathetic dystrophy. There was no conclusive evidence in the literature regarding any correlation between the treatment method used (surgical or conservative treatment) and higher frequency of any specific type of complication. It took six to 16 weeks for most of the interviewees to be able to return to work, both with conservative intervention (84%) and with surgical intervention (88%).

## CONCLUSIONS

There was a consensus between most of the interviewees regarding the following aspects of treating fractures of the distal radius: the three main factors in making decisions about the treatment type to use (whether the fracture was intra-articular, the presence of shortening of the distal radius and the patient's age); the immobilization type used for conservative treatment (above-elbow plaster immobilization); the fracture reduction method (manual reduction); the anesthesia type (local); the immobilization position (palmar flexion, ulnar deviation and neutral pronation/supination) and the use of bone graft (only in special cases).

There were conflicting opinions, without consensus, regarding the following: fracture classification method; surgical intervention method; most frequent complications; most frequently diagnosed lesion; and time taken to return to habitual activities.

### Implications for Practice and Research

Given the results from the present study and the best evidence from the literature, we conclude that there is no scientific evidence powerful enough to allow definitive conclusions concerning the main aspects of managing distal radius fractures. Trials using carefully designed methodology should be conducted in the future, in order to obtain high-quality evidence regarding classification systems, best methods for conservative and surgical treatment and criteria for defining instability patterns.

## References

[1] Handoll HH, Madhok R (2003). Surgical interventions for treating distal radial fractures in adults. Cochrane Database Syst Rev.

[2] Handoll HH, Madhok R (2003). Conservative interventions for treating distal radial fractures in adults. Cochrane Database Syst Rev.

[3] Handoll HH, Madhok R, Dodds C (2002). Anaesthesia for treating distal radial fracture in adults. Cochrane Database Syst Rev.

[4] Handoll HH, Madhok R, Howe TE (2002). Rehabilitation for distal radial fractures in adults. Cochrane Database Syst. Rev.

[5] O’Neill TW, Cooper C, Finn JD (2001). Incidence of distal forearm fracture in British men and women. Osteoporos Int.

[6] Nguyen TV, Center JR, Sambrook PN, Eisman JA (2001). Risk factors for proximal humerus, forearm, and wrist fractures in elderly men and women: the Dubbo Osteoporosis Epidemiology Study. Am J Epidemiol.

[7] Fernandez DL, Palmer AK, Green DP, Hotchkiss RN, Pederson WC (1999). Fractures of the distal radius. Green's operative hand surgery.

[8] Kapoor H, Agarwal A, Dhaon BK (2000). Displaced intra-articular fractures of distal radius: a comparative evaluation of results following closed reduction, external fixation and open reduction with internal fixation. Injury.

[9] Altissimi M, Mancini GB, Azzara A, Ciaffoloni E (1994). Early and late displacement of fractures of distal radius. The prediction of instability. Int Orthop.

[10] Lafontaine M, Hardy D, Delince P (1989). Stability assessment of distal radius fractures. Injury.

[11] Jupiter JB, Fernandez DL (1997). Comparative classification for fractures of the distal end of the radius. J Hand Surg [Am].

[12] Kreder HJ, Hanel DP, McKee M, Jupiter J, McGillivary G, Swiontkowski MF (1996). Consistency of AO fracture classification for the distal radius. J Bone Joint Surg Br.

[13] Cummings SR, Kelsey JL, Nevitt MC, O’Dowd KJ (1985). Epidemiology of osteoporosis and osteoporotic fractures. Epidemiol Rev.

[14] Earnshaw SA, Aladin A, Surendran S, Moran CG (2002). Closed reduction of colles fractures: comparison of manual manipulation and finger-trap traction: a prospective, randomized study. J Bone Joint Surg Am.

